# Selective activation of G_s_ signaling in adipocytes causes striking metabolic improvements in mice

**DOI:** 10.1016/j.molmet.2019.06.018

**Published:** 2019-06-20

**Authors:** Lei Wang, Sai P. Pydi, Yinghong Cui, Lu Zhu, Jaroslawna Meister, Oksana Gavrilova, Rebecca Berdeaux, Jean-Philippe Fortin, Kendra K. Bence, Cecile Vernochet, Jürgen Wess

**Affiliations:** 1 Molecular Signaling Section, Laboratory of Bioorganic Chemistry, National Institute of Diabetes and Digestive and Kidney Diseases, Bethesda, MD, 20892, USA; 2 Mouse Metabolism Core, National Institute of Diabetes and Digestive and Kidney Diseases, Bethesda, MD, 20892, USA; 3 Department of Integrative Biology and Pharmacology and Center for Metabolic and Degenerative Diseases at the Brown Foundation Institute of Molecular Medicine, McGovern Medical School at The University of Texas Health Science Center at Houston (UTHealth) and Graduate Program in Biochemistry and Cell Biology, MD Anderson Cancer Center-UTHealth Graduate School of Biomedical Sciences, Houston, TX, 77030, USA; 4 Internal Medicine Research Unit, Worldwide Research, Development and Medical, Pfizer Inc, Cambridge, MA, 02139, USA

**Keywords:** G protein-coupled receptor, G protein, DREADD technology, Adipocytes, Glucose homeostasis, Obesity

## Abstract

**Objective:**

Given the worldwide epidemics of obesity and type 2 diabetes, novel antidiabetic and appetite-suppressing drugs are urgently needed. Adipocytes play a central role in the regulation of whole-body glucose and energy homeostasis. The goal of this study was to examine the metabolic effects of acute and chronic activation of G_s_ signaling selectively in adipocytes (activated G_s_ stimulates cAMP production), both in lean and obese mice.

**Methods:**

To address this question, we generated a novel mutant mouse strain (adipo-GsD mice) that expressed a G_s_-coupled designer G protein-coupled receptor (Gs DREADD or short GsD) selectively in adipocytes. Importantly, the GsD receptor can only be activated by administration of an exogenous agent (CNO) that is otherwise pharmacologically inert. The adipo-GsD mice were maintained on either regular chow or a high-fat diet and then subjected to a comprehensive series of metabolic tests.

**Results:**

Pharmacological (CNO) activation of the GsD receptor in adipocytes of adipo-GsD mice caused profound improvements in glucose homeostasis and protected mice against the metabolic deficits associated with the consumption of a calorie-rich diet. Moreover, chronic activation of G_s_ signaling in adipocytes led to a striking increase in energy expenditure and reduced food intake, resulting in a decrease in body weight and fat mass when mice consumed a calorie-rich diet.

**Conclusion:**

Systematic studies with a newly developed mouse model enabled us to assess the metabolic consequences caused by acute or chronic activation of G_s_ signaling selectively in adipocytes. Most strikingly, chronic activation of this pathway led to reduced body fat mass and restored normal glucose homeostasis in obese mice. These findings are of considerable relevance for the development of novel antidiabetic and anti-obesity drugs.

## Highlights

• A novel mouse model allowed us to study the metabolic effects caused by activating G_s_ signaling selectively in adipocytes.
• Acute activation of this pathway caused numerous beneficial metabolic effects including improved glucose tolerance.
• Chronic activation of adipocyte G_s_ signaling reduced body fat mass and restored normal glucose homeostasis in obese mice.
• This anti-obesity effect was caused by a combination of enhanced energy expenditure and reduced calorie intake.
• These findings should stimulate the development of novel drugs useful for the treatment of obesity and type 2 diabetes.

## 1 Introduction

Type 2 diabetes (T2D) has emerged as a major health problem in most parts of the world. The T2D epidemic is fueled by the rapid rise in the prevalence of obesity, due to changes in lifestyle and diet [1], [2], [3], [4]. Since lasting changes in lifestyle and diet are difficult to achieve, there is a clear need for new antidiabetic drugs endowed with increased efficacy and reduced incidence of side effects.

Like most other cell types, adipocytes express many G protein-coupled receptors (GPCRs) which are present on the cell surface and can be activated by extracellular ligands such as hormones or neurotransmitters [5], [6]. Each GPCR displays a distinct G protein coupling preference, activating either G_s_- G_i_-, or G_q_-type G proteins which are linked to specific signaling pathways or networks and are predicted to have multiple effects on adipocyte function [6], [7]. At present, little is known about how activation of these various GPCR/G protein pathways affects glucose homeostasis under physiological and pathophysiological conditions in vivo. Studies in this field have been hampered by the lack of GPCR subtype-selective ligands and the fact that the GPCRs expressed by adipocytes are also present in many other tissues.

To circumvent these difficulties, we started to employ a novel experimental strategy that involves the use of designer GPCRs that can only be activated by an exogenously administered drug [8], [9]. These new designer receptors are generally referred to as DREADDs (Designer Receptors Exclusively Activated by a Designer Drug) [8], [9]. Most DREADDs are mutant muscarinic acetylcholine receptors that no longer respond to the endogenous ligand, acetylcholine, but can be activated by nanomolar concentrations of clozapine-N-oxide (CNO), a clozapine metabolite that has little or no pharmacological activity [8], [9].

We recently developed a mouse strain in which the expression of a G_s_-coupled DREADD (GsD) [10] can be induced in specific cell types in a Cre-dependent fashion [11]. In the present study, we examined the metabolic effects of acute and chronic activation of G_s_ signaling selectively in adipocytes, both in lean and obese mice. To address this question, we used the newly developed mouse strain described by Akhmedov et al. [11] to generate mutant mice that express the GsD receptor selectively in adipocytes (adipo-GsD mice).

Systematic metabolic studies with adipo-GsD mice showed that selective activation of a G_s_-coupled receptor in adipocytes causes profound improvements in glucose tolerance and insulin sensitivity and protects against the metabolic deficits associated with the consumption of a calorie-rich diet. Moreover, chronic activation of G_s_ signaling in adipocytes leads to a striking increase in energy expenditure and a decrease in food intake, leading to a significant loss in body weight when mice are maintained on a high-fat diet (HFD). These findings should be of considerable relevance for the development of novel antidiabetic and anti-obesity drugs.

## 2 Material and methods

### 2.1 Animals

We generated mutant mice that express the GsD receptor selectively in adipocytes (adipo-GsD mice) by crossing *ROSA26-LSL-GsDREADD-CRE-luc* mice [11] with *adipoq-Cre* mice (The Jackson Laboratory; stock no. 010803; genetic background: C57BL/6J). The *ROSA26-LSL-GsDREADD-CRE-luc* mice used for these matings had been backcrossed for 10 generations onto a C57BL/6 background. *ROSA26-LSL-GsDREADD-CRE-luc* mice that lacked the *adipoq-Cre* transgene served as control animals. All experiments were carried out with male littermates. Mice were housed under conditions of controlled temperature (23 °C) and illumination (12-h light/12-h dark cycle, light off at 6 pm), and had free access to water and food. Mice consumed standard mouse chow (7022 NIH-07 diet, 15% kcal fat, energy density 3.1 kcal/g, Envigo Inc.) or were switched to a high-fat diet (HFD; F3282, 60% kcal fat, energy density 5.5 kcal/gm, Bioserv) when they were 6 weeks old. Unless stated otherwise, mice were maintained on the HFD diet for at least 8 weeks.

All animal studies were carried out according to the US National Institutes of Health Guidelines for Animal Research and were approved by the NIDDK Institutional Animal Care and Use Committee.

### 2.2 In vivo metabolic tests

In vivo metabolic tests were carried out with male mice (8–20 weeks of age) using standard procedures. Intraperitoneal glucose tolerance tests (IGTT) were performed with mice that had been fasted overnight for about 16 h. Blood glucose concentrations were determined before and after i.p. injection with glucose (1 or 2 g/kg, as indicated). Blood glucose concentrations were determined by collecting blood from the tail vein using a portable glucometer (Glucometer Contour; Bayer). For insulin tolerance tests (ITT), mice that had been fasted for 4 h were injected i.p. with human insulin (0.75 or 1 U/kg, as indicated; Humulin, Eli Lilly). To study glucose-stimulated insulin secretion (GSIS), mice that had been fasted overnight were injected i.p. with 1 or 2 g/kg of glucose, as indicated. Plasma insulin levels were detected by using a mouse insulin ELISA kit (Crystal Chem Inc.).

For acute CNO challenge tests, mice that had been fasted for 4 h were injected i.p. with 10 mg/kg CNO. Blood was collected from the tail vein before and at specific time points after CNO treatment. Plasma FFA levels were determined using a commercially available kit (Sigma–Aldrich). Plasma leptin and adiponectin levels were also measured by employing ELISA kits (R&D Systems).

For chronic CNO treatment experiments, mice that had been maintained on a HFD for 4 weeks received single daily injections of CNO (10 mg/kg i.p.) for 4 weeks. During the CNO injection period, mice continued to consume the same HFD as before.

### 2.3 Body composition analysis

The lean/fat mass composition of mice was measured using the 3-in-1 Echo MRI Composition Analyzer (Echo Medical System).

### 2.4 Real-time qPCR gene expression analysis

Mouse tissues were collected and frozen quickly. Total mRNA was extracted and purified by using the RNeasy mini kit combined with the RNase-free DNase set (Qiagen), following the manufacturer's protocol. cDNA was synthesized using SuperScript™ III First-Strand Synthesis SuperMix (Invitrogen). Real-time qPCR was performed using both the SYBR Green and TaqMan methods (Applied Biosystems). TaqMan primer/probe sets for real-time PCR were designed using Primer Express software (Applied Biosystems) and were purchased from Integrated DNA Technologies. RNA expression data were normalized relative to the expression of β-actin or 18S rRNA. The PCR primer sequences and probes are listed in Supplemental Table 1.

### 2.5 Histology

Adipose and liver tissues were fixed in 4% paraformaldehyde for 24 h and embedded in Optimal cutting temperature compound (OCT). Five-μm-thick sections were prepared, and sections were mounted and then stained with hematoxylin and eosin (H&E) and/or Oil Red O. Bright-field images of stained tissue sections were taken with the Keyence Microscope BZ-9000.

### 2.6 Indirect calorimetry and energy expenditure measurements

Indirect calorimetry and energy expenditure measurements were performed using Oxymax-CLAMS (Columbus Instruments) chambers [12], [13]. Mice maintained on regular chow were acclimatized to the chambers for 2 days at 30 °C (thermoneutrality) and then received a single injection of CNO (10 mg/kg i.p.) or vehicle (saline). For each mouse, food intake, O_2_ consumption, CO_2_ production, and ambulatory activity (infrared beam breaks) were determined every 13 min for 6.5 h (390 min). Total energy expenditure (TEE) and respiratory exchange ratio (RER) were calculated based on O_2_ consumption and CO_2_ production. Mice maintained on a high-fat diet (HFD) were treated in a similar fashion, except that they received daily CNO injections (10 mg/kg i.p.) for one week. Indirect calorimetry studies were also carried out with mice housed at room temperature (22 °C), following a 2-day acclimatization period.

### 2.7 Cold tolerance test

Mice that had been housed at room temperature were transferred to 4 °C, and rectal temperature was measured hourly for up to 6 h.

### 2.8 Liver triglyceride content

Livers were homogenized in PBS, and protein concentrations determined. Total lipid was extracted from the homogenate with chloroform/methanol (2:1). An aliquot of the organic phase was collected, dried overnight, and re-suspended in 1% Triton X-100 EtOH. Hepatic triglyceride content was determined using a commercially available kit (Sigma Aldrich).

### 2.9 cAMP assay

cAMP levels in mouse adipose tissue (iWAT) were measured using a cAMP ELISA kit (Cayman Chemical). Briefly, iWAT was rapidly collected, homogenized, and extracted, followed by the detection of cAMP in the supernatant according to the manufacturer's protocol. Protein concentrations were determined using a bicinchoninic acid assay kit (Pierce).

### 2.10 Isolation of mature adipocytes

Adipose tissue (iWAT and eWAT) of 16-week-old C57BL/6J mice (males) was digested with KRH buffer (2% FFA-free BSA) containing 3.3 mg/ml collagenase via incubation at 37 °C for 30–45 min. When the tissue was fully digested, 10 ml of KRH buffer was added to terminate collagenase activity. Cells were then filtered through a 250 μm cell strainer. After 10 min, the floating top layer containing mature adipocytes was collected. Mature adipocytes were washed twice with KRH buffer containing 5 mM EDTA and then used for RNA-seq analysis.

### 2.11 RNA-seq study

Total RNA extracted from mature adipocytes (iWAT and eWAT) and BAT tissue of C57BL/6J mice (16-week old males) maintained on regular chow were used to construct high throughput sequencing libraries. RNAs with RIN >8 (assessed by the Agilent 2100 Bioanalyzer system) were used to prepare transcriptome libraries using the NEBNext Ultra RNA library prep kit (New England Biolabs). High throughput RNA-sequencing was performed using a HiSeq 2500 instrument (Illumina) at the NIDDK Genomic Core Facility (NIH, Bethesda, MD). The raw expression data were analyzed by the Genomatix Genome Analyzer. Raw reads were mapped to the mouse (mm9) genome. GPCRs were extracted from the RNA-seq data using R form (http://www.R-project.org/). RPKM data are given as mean values (n = 6). G_s_-coupled GPCRs were identified using the IUPHAR/BPS Guide to Pharmacology Database (https://www.guidetopharmacology.org/). The RNA-seq data are viewable under GEO accession number GSE131861.

### 2.12 Statistics

All data are expressed as mean ± SEM. The Shapiro–Wilk normality test was performed to determine whether the data were normally distributed. Data were then tested for statistical significance by one- or two-way ANOVA, followed by the indicated post hoc tests, or by using a two-tailed unpaired Student's t test, as appropriate. *P* < 0.05 was considered statistically significant. The specific statistical tests that were used are indicated in the figure legends.

## 3 Results

### 3.1 Generation of adipo-GsD mice

To generate mutant mice that express the GsD receptor selectively in adipocytes (adipo-GsD mice), we crossed *ROSA26-LSL-GsDREADD-CRE-luc* mice [11] with *adipoq-Cre* mice, which express Cre recombinase under the control of the adipocyte-specific *adiponectin* promoter [14]. Taqman qPCR analysis showed that the GsD receptor was selectively expressed in mouse adipose tissues (Supplemental Fig. 1A).

### 3.2 Metabolic studies with mice maintained on regular chow

**Figure 1:**
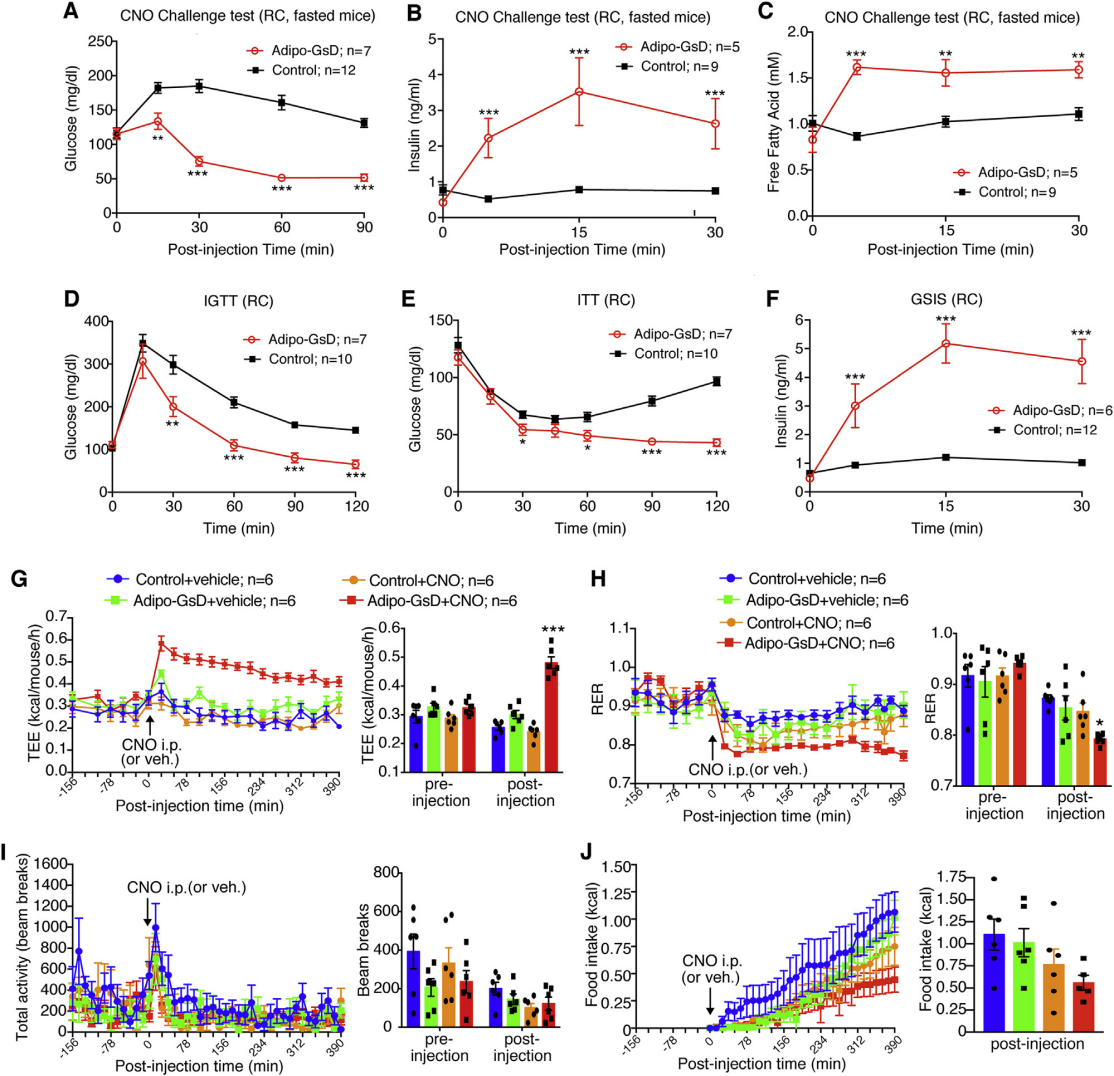
Acute activation of adipocyte G_s_ signaling improves glucose metabolism and increases energy expenditure in mice consuming regular chow. (A–F) In vivo metabolic tests were carried out with adipo-GsD mice and control littermates consuming regular mouse chow (RC). (A–C) CNO challenge tests. Mice that had been fasted overnight were injected with CNO (10 mg/kg, i.p.), and blood glucose levels (A), plasma insulin levels (B), and plasma free fatty acid (FFA) levels (C) were measured at the indicated time points. (D) Intraperitoneal glucose tolerance test (IGTT, 2 g glucose/kg, i.p.). (E) Insulin tolerance test (ITT, 0.75 U/kg, i.p.). (F) Glucose-stimulated insulin secretion (GSIS, 2 g glucose/kg, i.p.). (G–J) Indirect calorimetry studies were carried out with RC mice before and after acute injection of CNO (10 mg/kg, i.p.) or vehicle. Mice were housed in Oxymax/CLAMS chambers at 30 °C (thermoneutrality). Total energy expenditure (TEE) (G), respiratory exchange ratio (RER) (H), total locomotor activity (I), and cumulative food intake (J) were monitored. The bar diagrams show average values calculated from all pre- and post-injection measurements. All studies were performed with 8–12-week-old male mice. Data represent mean ± s.e.m. (mouse numbers are indicated in each panel). *P < 0.05, **P < 0.01, and ***P < 0.001 vs. control. Statistical significance was determined by two-way ANOVA followed by Bonferroni's post-hoc test (A–F) or by one-way ANOVA followed by Bonferroni's post-hoc test (G–J).

We subjected adipo-GsD mice and their control littermates (*ROSA26-LSL-GsDREADD-CRE-luc* mice lacking the Cre transgene) consuming regular mouse chow (RC) to a series of metabolic tests. Prior to CNO administration, adipo-GsD and control mice showed no significant differences in body weight (Supplemental Fig. 1B), as well as fed and fasting blood glucose and plasma insulin levels (Supplemental Figs. 1C and D). To study the metabolic effects of acute activation of G_s_ signaling in mouse adipocytes in vivo, we injected adipo-GsD and control mice with a single dose of CNO (10 mg/kg i.p.) (CNO challenge test). The acute CNO injection caused an increase in blood glucose levels in the control animals (Figure 1A), most likely caused by the injection stress. In striking contrast, acute CNO treatment of adipo-GsD mice led to a pronounced and long-lasting decrease in blood glucose levels (Figure 1A). As expected, CNO treatment increased cAMP levels in fat tissue (iWAT) prepared from adipo-GsD mice, as compared to iWAT from control littermates (Supplemental Fig. 1E).

We also monitored CNO-induced changes in plasma insulin levels. While acute CNO (10 mg/kg i.p.) treatment of control mice had no significant effect on plasma insulin levels, CNO caused a robust increase in plasma insulin levels in adipo-GsD mice (Figure 1B). Moreover, CNO treatment of adipo-GsD mice, but not of control littermates, triggered a significant increase in plasma free fatty acid (FFA) levels, consistent with the known ability of G_s_ signaling to trigger PKA-dependent lipolysis [15].

Importantly, CNO-treated adipo-GsD mice also showed significant improvements in glucose tolerance (Figure 1D), whole-body insulin sensitivity (Figure 1E), and glucose-stimulated insulin-secretion (GSIS) (Figure 1F), as compared to their control littermates.

### 3.3 Indirect calorimetry studies with mice maintained on RC

Since adipose tissue plays a central role in the regulation of energy homeostasis [16], [17], [18], [19], we studied total energy expenditure (TEE) via indirect calorimetry. All measurements were carried out at thermoneutrality (30 °C) since mouse studies carried out under these conditions more closely mimic human physiology [20]. Acute CNO (10 mg/kg i.p.) treatment of adipo-GsD mice maintained on RC caused a very robust and long-lasting (>6 h) increase in TEE (Figure 1G). CNO treatment of control mice or saline (vehicle) treatment of adipo-GsD or control mice had no significant effect on TEE (Figure 1G). CNO-treated adipo-GsD mice also showed a significant reduction in the respiratory exchange ratio (RER), as compared to the three control groups (Figure 1H), most likely due to elevated plasma FFA levels (Figure 1C). The four groups of mice did not differ in total locomotor activity, either before or after CNO treatment (Figure 1I). CNO-treated adipo-GsD mice showed a trend towards reduced food intake, bust this effect failed to reach statistical significance (Figure 1J).

### 3.4 Metabolic studies with mice consuming an obesity-inducing diet

**Figure 2:**
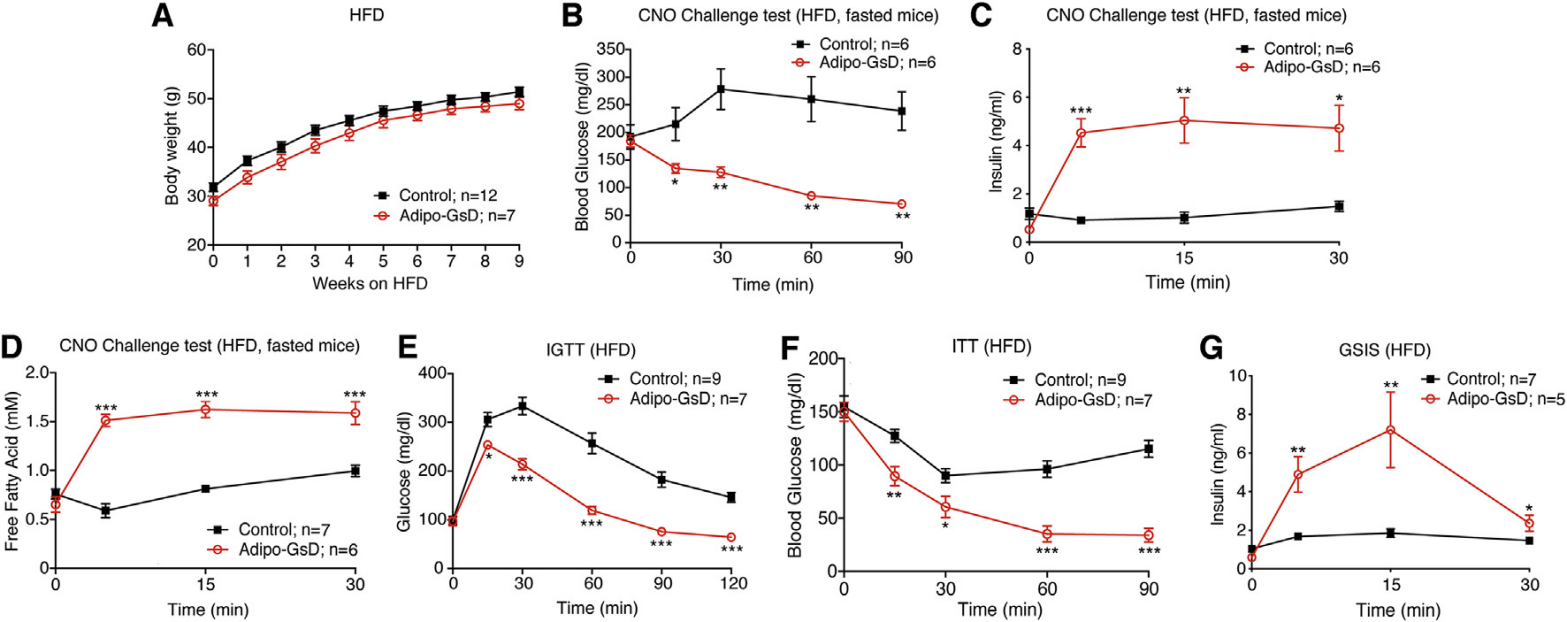
Acute activation of adipocyte G_s_ signaling improves glucose homeostasis in obese mice. (A) Body weight gain of adipo-GsD and control mice maintained on a high-fat diet (HFD). (B–D) CNO challenge tests. HFD mice that had been fasted overnight were injected with CNO (10 mg/kg, i.p.), and blood glucose levels (B), plasma insulin levels (C), and plasma FFA levels (D) were measured at the indicated time points. (E) Intraperitoneal glucose tolerance test (IGTT, 1 g glucose/kg, i.p.). (E) Insulin tolerance test (ITT, 1 U/kg, i.p.). (F) Glucose-stimulated insulin secretion (GSIS, 1 g glucose/kg, i.p.). Data represent mean ± s.e.m. (mouse numbers are indicated in each panel). The experiments shown in (B–G) were obtained with male mice (littermates) that had been maintained on the HFD for at least 8 weeks. *P < 0.05, **P < 0.01, and ***P < 0.001 vs. control. Statistical significance was determined by two-way ANOVA followed by Bonferroni's post-hoc test.

We next carried out metabolic studies with mice that had been maintained on a high-fat diet (HFD) for at least 8 weeks (Figure 2A). The HFD causes obesity and all major metabolic deficits characteristic for T2D including glucose intolerance and insulin resistance [21]. When consuming the HFD, adipo-GsD and control mice showed similar body weight gain (Figure 2A). After 8 weeks of HFD feeding, we monitored CNO-induced changes in blood glucose, plasma insulin, and plasma FFA levels. As observed with RC adipo-GsD mice (Figure 1A–C), CNO (10 mg/kg i.p.) treatment of HFD adipo-GsD mice caused pronounced decreases in blood glucose levels, accompanied by striking increases in plasma insulin and FFA levels (Figure 2B–D). Acute CNO administration also greatly improved glucose tolerance, insulin sensitivity, and GSIS in the HFD adipo-GsD mice (Figure 2E–G).

### 3.5 Chronic CNO treatment of HFD mice

**Figure 3:**
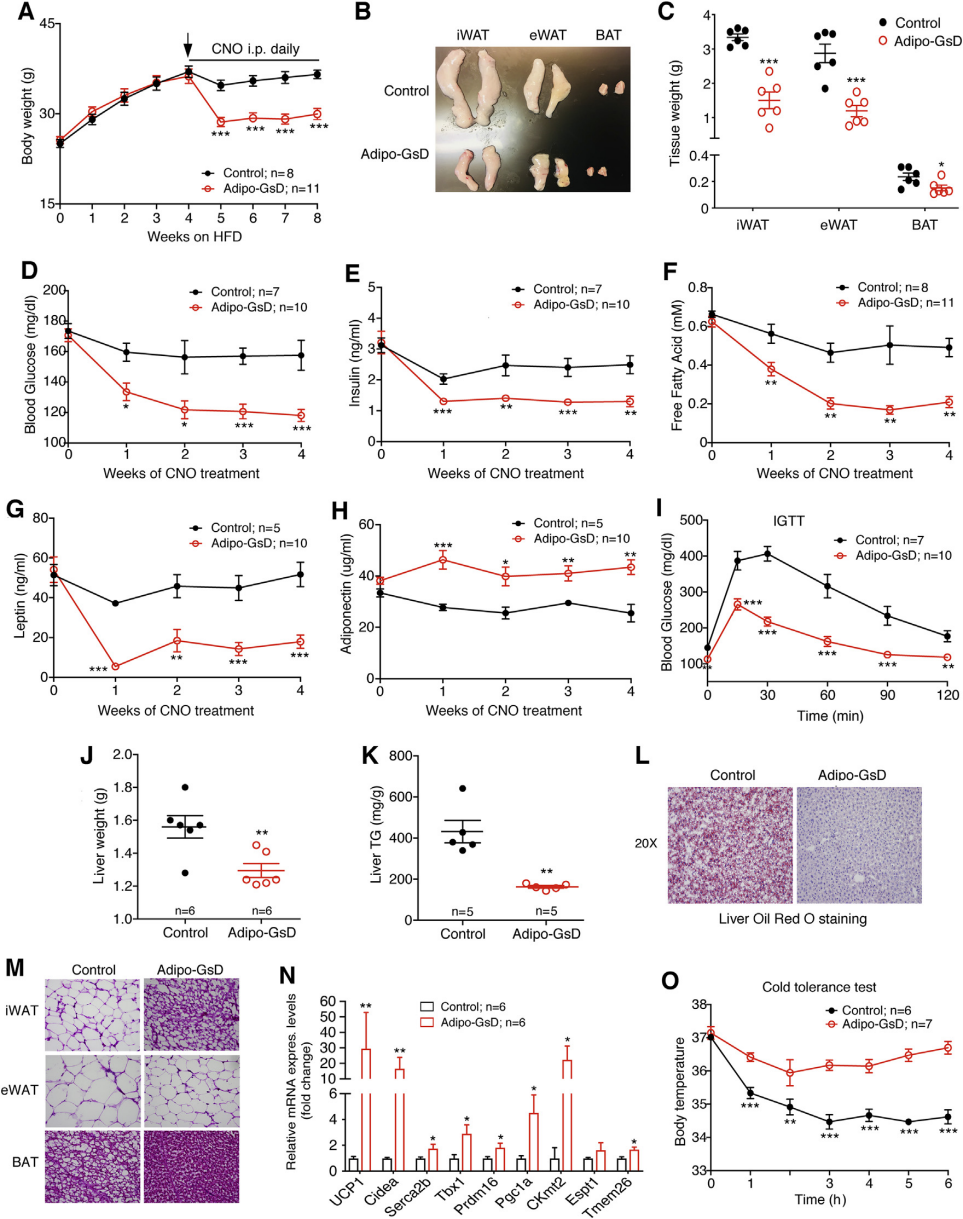
Chronic activation of adipocyte G_s_ signaling significantly decreases body weight and improves glucose homeostasis in mice consuming a HFD. All studies shown in this figure were carried out with male adipo-GsD and control mice that had been maintained on a HFD for 8 weeks and had received daily injections of CNO (10 mg/kg, i.p.) during the last 4 weeks of HFD feeding. (A) Body weight gain of HFD adipo-GsD and control mice chronically treated with CNO. (B) Representative photographs of inguinal WAT (iWAT), epididymal WAT (eWAT), and brown adipose tissue (BAT) following chronic CNO treatment. (C) Tissue weight of iWAT, eWAT and BAT after chronic CNO treatment. (D) Blood glucose, (E) plasma insulin, (F) plasma FFA, (G) plasma leptin, and (H) plasma adiponectin levels before and after chronic CNO administration. (I) Intraperitoneal glucose tolerance test (IGTT, 1 glucose/kg, i.p.) carried out after chronic CNO treatment. (L) Liver weight and (M) liver triglyceride (TG) content of mice following chronic CNO administration. (N) Images of liver sections stained with Oil Red O after chronic CNO treatment. (M) H&E staining of adipose tissues after chronic CNO treatment. (N) Expression of beiging-related genes in iWAT following chronic CNO administration. (O) Cold tolerance test (4 °C) after chronic CNO treatment. All studies were performed with male littermates. Data represent mean ± s.e.m. (mouse numbers are indicated in each panel). *P < 0.05, **P < 0.01, and ***P < 0.001 vs. control. Significance was determined by two-way ANOVA followed by Bonferroni's post-hoc test (A, D-I, O) or by two-tailed Student's t test (C, J, K, and N).

We next studied the effect of chronic CNO treatment on HFD adipo-GsD and control mice. After four weeks of HFD feeding, we treated both groups of mice with daily injections of CNO (10 mg/kg i.p.) for 4 weeks. During this time, the mice continued to consume the HFD. Chronic CNO treatment led to a significant and sustained loss in body weight in the HFD adipo-GsD mice, but not in the HFD control littermates (Figure 3A). Consistent with this observation, the mass of inguinal white adipose tissue (iWAT), epididymal WAT (eWAT), and brown adipose tissue (BAT) was greatly reduced in the HFD adipo-GsD mice at the end of the 4-week CNO treatment period (Figure 3B,C). Chronic CNO treatment of HFD adipo-GsD mice led to significant reductions in blood glucose, plasma insulin, plasma FFA, and plasma leptin levels, but increased plasma adiponectin levels (Figure 3D–H). Moreover, CNO-treated HFD adipo-GsD mice displayed a striking improvement in glucose tolerance (Figure 3I), and a pronounced reduction in liver weight (Figure 3J) and hepatic triglyceride (lipid) content (Figure 3K,L).

In H&E staining experiments, iWAT from CNO-treated HFD adipo-GsD mice showed a staining pattern typical for the appearance of beige fat (Figure 3M). In agreement with this observation, qRT-PCR studies showed that the expression of key genes involved in iWAT beiging (*Ucp1*, *Cidea*, etc.) was significantly upregulated in CNO-treated HFD adipo-GsD mice (Figure 3N). Similarly, the transcript levels of several key mitochondrial marker genes were significantly increased in iWAT of CNO-treated HFD adipo-GsD mice (Supplemental Fig. 2). Finally, cold exposure (4 °C) of HFD adipo-GsD and control mice showed that chronic CNO treatment enabled the HFD adipo-GsD mice to maintain normal body temperature during the 6 h observation period (Figure 3O). In contrast, HFD control mice displayed significant hypothermia under the same experimental conditions (Figure 3O), indicative of increased thermogenic activity in BAT and iWAT of HFD adipo-GsD mice (Figure 3M, N).

**Figure 4:**
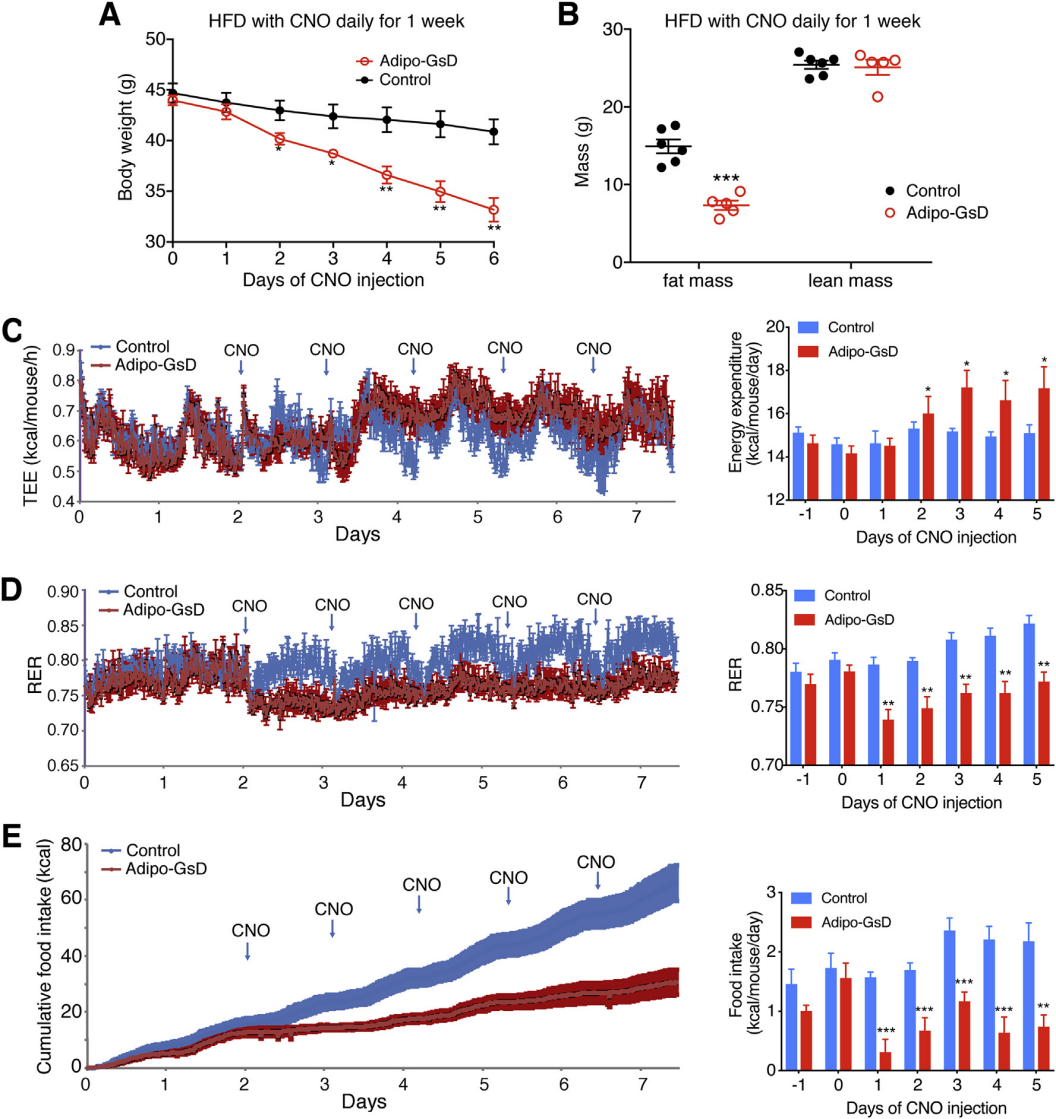
Chronic activation of adipocyte G_s_ signaling stimulates total energy expenditure and reduces food intake in HFD mice. All studies shown in this figure were carried out with male adipo-GsD and control mice that had been maintained on a HFD for 5 weeks and had received daily injections of CNO (10 mg/kg i.p.) during the last week of HFD feeding. In HFD adipo-GsD mice, daily CNO injections reduced body weight (A) and total fat mass (B). For indirect calorimetry studies (C–E), mice were housed in Oxymax/CLAMS chambers at room temperature (22 °C). HFD adipo-GsD mice showed increased total energy expenditure (TEE) (C), decreased respiratory exchange ratio (RER) (D), and reduced food intake (E), as compared to control littermates. All studies were performed using 8-12-week-old male littermates. Data represent mean ± s.e.m. (control, n = 6; adipo-GsD, n = 5). *P < 0.05, **P < 0.01, and ***P < 0.001 vs. control. Statistical significance was determined by two-way ANOVA followed by Bonferroni's post-hoc test (A) or by two-tailed Student's t test (B–E).

Figure 3A indicates that CNO treatment of HFD adipo-GsD mice for 1 week was sufficient to cause a major reduction in body weight. To explore the mechanisms underlying this phenotype, we carried out metabolic studies with adipo-GsD and control mice that had been maintained on a HFD for 5 weeks and had received daily injections of CNO (10 mg/kg i.p.) during the last week of HFD feeding. As expected, CNO treatment led to a time-dependent decrease in body weight in the HFD adipo-GsD mice (Figure 4A), accompanied by a ∼50% reduction in body fast mass (Figure 4B), as compared to HFD control littermates. In the HFD adipo-GsD mice, the daily CNO injections caused a significant increase in TEE (Figure 4C) and a decrease in RER (Figure 4D), as compared to the CNO-injected HFD control group. Moreover, the CNO-treated HFD adipo-GsD mice showed a dramatic decrease in daily food intake, as compared to the CNO-injected HFD control mice (Figure 4E). Total locomotor activity was not significantly different between the CNO-injected HFD adipo-GsD and control mice (Supplemental Fig. 3).

### 3.6 G_s_-linked GPCRs that are endogenously expressed by mouse adipocytes

To identify G_s_-linked GPCRs that are endogenously expressed by mouse adipose tissue, we subjected RNA prepared from isolated mouse adipocytes (iWAT and eWAT) and BAT tissue to RNA-seq analysis. This analysis demonstrated that mouse adipocytes/BAT tissue express several GPCRs that are selectively coupled to G_s_, including the V_2_ vasopressin receptor, the glucagon receptor, and different melanocortin receptor subtypes (Supplemental Table 2).

## 4 Discussion

In this study, we used DREADD technology to study the metabolic consequences of selective activation of G_s_ signaling in mouse adipose tissues. Specifically, we generated a novel mutant mouse strain that expresses a G_s_-coupled designer GPCR (GsD) selectively in adipose tissues (adipo-GsD mice). In these mice, G_s_ signaling can be selectively stimulated in adipose tissues in a drug (CNO)-dependent fashion.

Independent of the diet that the mice consumed (RC or HFD), acute CNO treatment of adipo-GsD mice caused pronounced, rapid improvements in glucose tolerance (Figure 1, Figure 2E), most likely due to enhanced insulin release (Figure 1, Figure 2C). As expected, CNO treatment of adipo-GsD mice stimulated lipolysis, resulting in enhanced plasma FFA levels (Figure 1, Figure 2D). Increased plasma FFA levels are predicted to promote the release of insulin by stimulating the FFA-responsive GPR40 receptor (FFA1 receptor) expressed in pancreatic β-cells [22], [23], [24], suggesting a likely mechanism underlying the ability of CNO to increase insulin release in adipo-GsD mice.

Chronic CNO treatment of mice maintained on a HFD yielded a series of striking metabolic phenotypes (Figure 3, Figure 4). Under these experimental conditions, CNO caused a robust reduction in body weight and obesity, associated with greatly improved glucose tolerance and reduced hepatic fat accumulation (Figure 3, Figure 4A, B). Moreover, chronic CNO treatment of HFD adipo-GsD mice led to a significant increase in TEE and a reduction in food intake (Figure 4C,E), suggesting that the anti-obesity effect caused by chronic activation of adipocyte G_s_ signaling is due to a combination of enhanced energy expenditure and reduced caloric intake.

The observed increase in energy expenditure is most likely due to the increased thermogenic activity of BAT and iWAT caused by chronic CNO treatment of HFD adipo-GsD mice (Figure 3M−O). At present, the physiological basis underlying the appetite-suppressing effect of CNO in HFD adipo-GsD mice remains unclear. Interestingly, this phenotype was observed despite a pronounced reduction in plasma leptin levels (Figure 3G). One possibility is that this anorectic effect is caused by changes in the release of one or more hormones/adipokines from adipose tissues. It is also possible that CNO-stimulated thermogenesis in BAT and other fat depots activates an afferent neuronal signaling pathway that modulates feeding behavior in the brain. Clearly, these mechanistic issues need to be addressed in future studies.

The β3-adrenergic receptor (β3-AR) is the predominant β-AR subtype expressed by rodent adipocytes [25], [26]. Like the β1-and β2-ARs, the β3-AR is efficiently coupled to G_s_ [27]. Interestingly, several studies have shown that treatment of mice or rats with CL316,243 or other β3-AR-selective agonists causes anti-obesity and antidiabetic effects similar to those described here for CNO-treated adipo-GsD mice [20], [26], [28], [29], [30]. These effects are thought to be mediated by β3-ARs expressed by rodent adipocytes [31], [32].

Although the β3-AR is predominantly linked to G_s_, it can also activate G proteins of the G_i_ family [33], [34], [35]. In contrast, the GsD designer receptor used in the present study does not seem to couple to other G proteins besides G_s_ [8], [9], [10], suggesting that the metabolic responses observed after CNO stimulation of adipo-GsD mice are caused by enhanced adipocyte G_s_ signaling. In contrast, the physiological effects seen after treatment of rodents with CL316,243 or other β3-AR-selective agonists are likely due to the activation of both G_s_ and G_i_-type G proteins. For this reason, the metabolic phenotypes displayed by CNO-treated adipo-GsD mice can be interpreted in a more straightforward manner.

The use of adipo-GsD mice as a model system to study the metabolic consequences of activating G_s_ signaling in adipose tissue offers another major advantage. As shown in Supplemental Fig. 1A, the GsD designer receptor is selectively expressed in adipose tissues of adipo-GsD mice. In contrast, the β3-AR is not only expressed by adipocytes but also by many other cell types and tissues, including heart, brain, kidney, and urinary bladder [36], [37]. As a result, the metabolic effects observed after treatment of rodents with selective β3-AR agonists are most likely caused by the activation of both adipocyte- and non-adipocyte-β3-ARs.

In fact, several years ago, a selective β3-AR agonist, mirabegron, was approved for the treatment of overactive bladder, due to the presence of β3-ARs on bladder smooth muscle. A recent study reported that acute treatment of healthy male individuals with mirabegron increased resting metabolic rate, probably due to stimulation of β3-ARs expressed by human BAT [38]. However, it remains to be seen whether mirabegron or β3-AR agonists with increased selectivity and efficacy will prove useful for treating human metabolic disorders including obesity and T2D. In any case, the search for compounds that can stimulate G_s_ signaling in human adipocytes appears to be a very attractive goal.

In conclusion, the generation and use of a novel mouse model (adipo-GsD mice) allowed us to monitor the metabolic effects after acute or chronic activation G_s_ signaling selectively in adipocytes. Most strikingly, chronic activation of this pathway reduced body fat mass in obese mice and restored normal glucose homeostasis. The development of compounds that can selectively activate G_s_ signaling in adipocytes should prove highly beneficial for the treatment of obesity and T2D.

## Author contributions

L.W., C.V., and J.W. designed the experiments. L.W., S.P., Y.C., L.Z., and J.P.F. performed experiments and analyzed and interpreted the resulting data. O.G. provided guidance throughout this study and carried out the indirect calorimetry experiments. C.V., J.P.F., and K.B. gave helpful advice throughout this study. R.B. provided the *ROSA26-LSL-GsDREADD-CRE-luc* mice as a novel experimental tool. L.W. and J.W. wrote the manuscript.
