# Supplementary material for: Selective activation of Gs signaling in adipocytes causes striking metabolic improvements in mice
Source: Mol Metab. 2019 Jun 20;27:83–91. doi: 10.1016/j.molmet.2019.06.018 (PMC6717953; doi:10.1016/j.molmet.2019.06.018)
Supplement: Multimedia component 1 [file mmc1.pdf]

SUPPLEMENTAL MATERIAL:

Selective activation of G_s signaling in adipocytes causes striking metabolic improvements in mice

Lei Wang, Sai P. Pydi, Yinghong Cui, Lu Zhu, Jaroslawna Meister, Oksana Gavrilova, Rebecca Berdeaux, Jean-Philippe Fortin, Kendra K Bence, Cecile Vernochet, Jürgen Wess

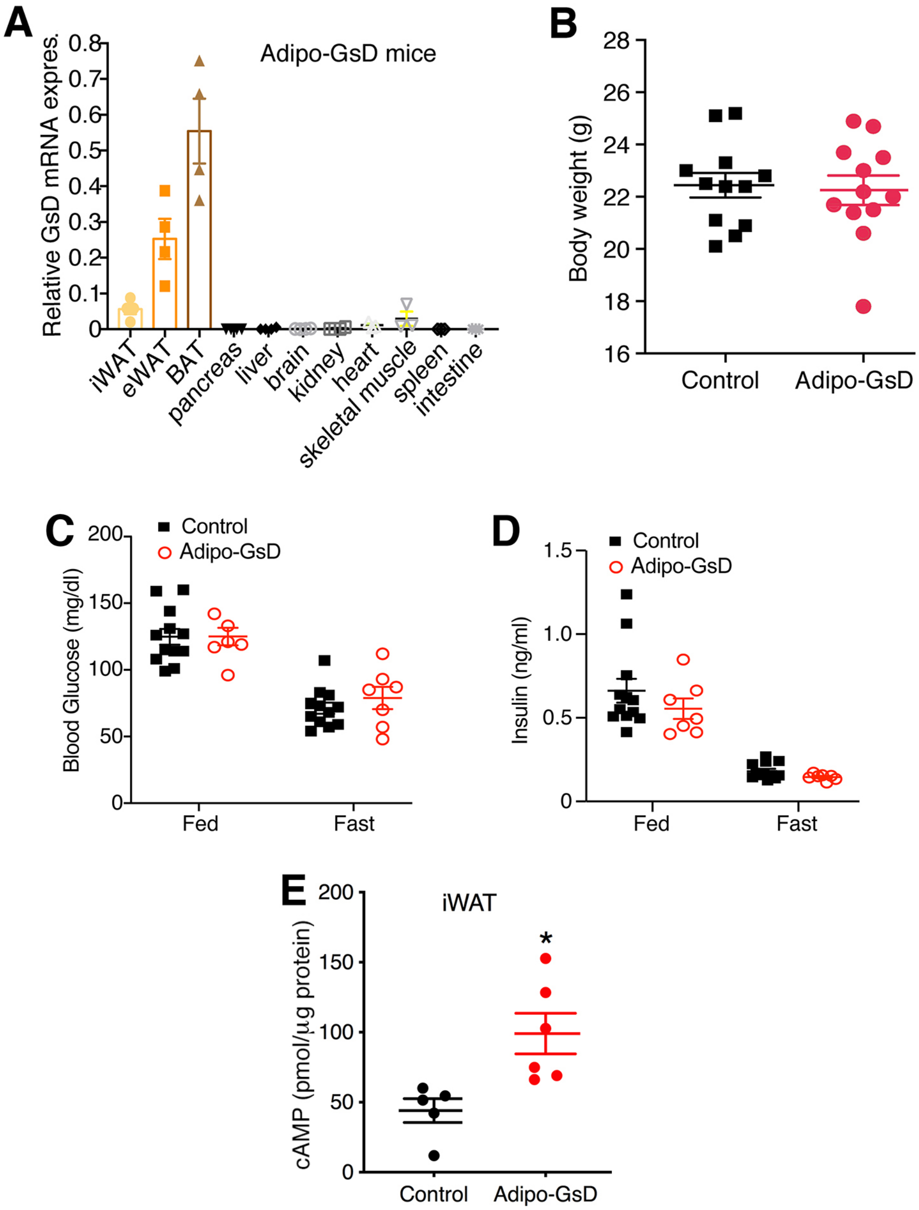

Supplemental Figure 1. Adipo-GsD mice exclusively express GsD in adipose tissues and show unchanged body weight, blood glucose, and plasma insulin levels. (A) Relative GsD mRNA expression levels in different tissues of adipo-GsD mice detected by Taqman qPCR (n=4). (B) Body weight of adipo-GsD mice and control littermates (males) maintained on regular chow (RC) for 8 weeks (n=12 per group). Blood glucose (C) and plasma insulin (D) levels (fed and fasted) of adipo-GsD mice and control littermates (males) maintained on RC for 8 weeks (control, n=12; adipo-GsD, n=7). (E) CNO-induced increase in cAMP levels in iWAT of adipo-GsD mice. Male adipo-GsD mice and control littermates maintained on RC were injected with CNO (10 mg/kg i.p.). Fifteen min later, mice were euthanized and iWAT cAMP levels were determined (n=6 per group). Data are given as means±s.e.m. Statistical significance was assessed by two-tailed Student's t test. *$P<0.05$, vs. control.

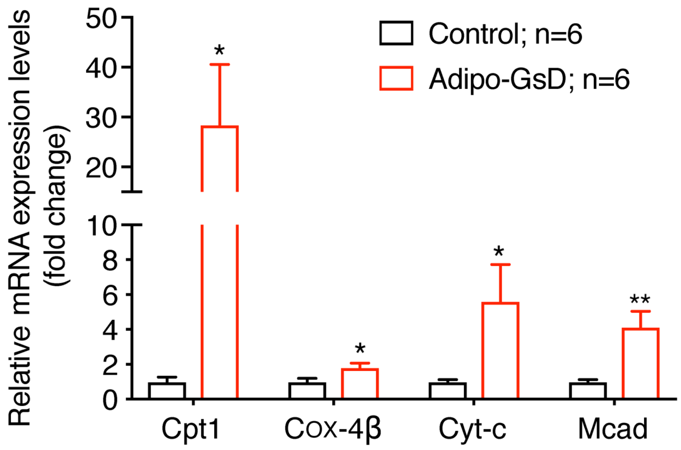

Supplemental Figure 2. Chronic activation of adipocyte G_s signaling promotes the expression of key genes involved in mitochondrial function. iWAT was prepared from male adipo-GsD and control mice that had been maintained on a HFD for 8 weeks and had received daily injections of CNO (10 mg/kg i.p.) during the last 4 weeks of HFD feeding. The expression levels of the indicated genes were studied by qRT-PCR. Cpt1, carnitine palmitoyltransferase 1; Cox-4β, cytochrome c oxidase subunit 4β; Cyt-c, mitochondrial cytochrome c; Mcad, medium-chain acyl-CoA dehydrogenase. Data represent means±s.e.m. Statistical significance was assessed by two-tailed Student's t test. *P<0.05, **P<0.01, vs. control.

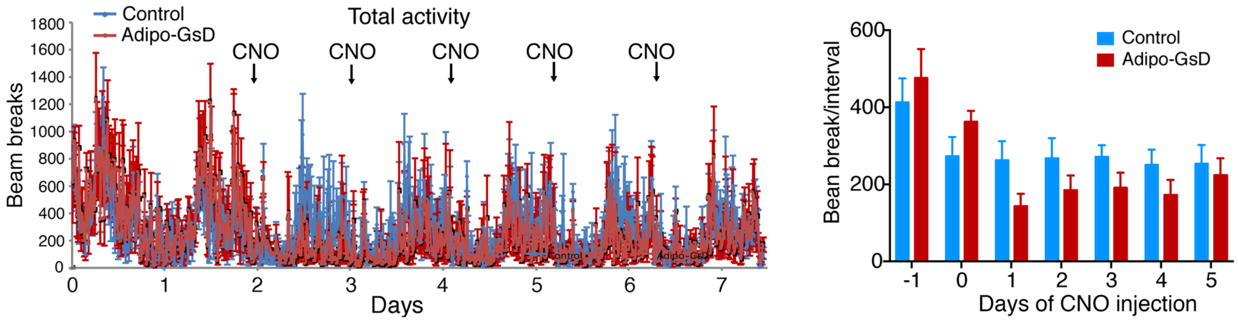

Supplemental Figure 3. Total locomotor activity is not significantly affected by chronic CNO treatment of HFD adipo-GsD mice. Locomotor activity measurements were carried out with male adipo-GsD and control mice that had been maintained on a HFD for 8 weeks and had received daily injections of CNO (10 mg/kg i.p.) during the last week of HFD feeding. Data represent mean± s.e.m. (control, n=6; adipo-GsD, n=5).

Supplemental Table 1. List of primers/probes used for gene expression analysis

Gene Target	Species	Primer sequence
GsD	mouse	Probe: 5'-/56-FAM/TTCTCCTCA/ZEN/AACGACACCTCCAGC/3IABkFQ/-3'<br>Primer 1: 5'-GTTGGGCAGCTACAACATTTC-3'<br>Primer 2: 5'-ATGCCAGGAAGCCAGTTAAG-3' (for Taqman analysis)
β-actin	mouse	Probe: 5'-/56-FAM/TGGCATTGT/ZEN/TACCAACTGGGACGA/3IABkFQ/-3'<br>Primer 1: 5'-GAGGTATCCTGACCCTGAAGTA-3'<br>Primer 2: 5'-CACACGCAGCTCATTGTAGA-3' (for Taqman analysis)
β-actin	mouse	Forward: 5'-GATATCGCTGCGCTGGTCGTC-3'<br>Reverse: 5'-ACGCAGCTCATTGTAGAAGGTGTGG-3'<br>(for SYBR Green analysis)
Ucp-1	mouse	Forward: 5'-ACTGCCACACCTCCAGTCATT-3'<br>Reverse: 5'-CTTTGCCTCACTCAGGATTGG-3'
18S rRNA	mouse	Forward: 5'-CGGCTACCACATCCAAGGAA-3'<br>Reverse: 5'-GCTGGAATTACCGCGGCT-3'
Cidea	mouse	Forward: 5'-TGCTCTTCTGTATCGCCCAGT-3'<br>Reverse: 5'-GCCGTGTTAAGGAATCTG CTG-3'
Serca2b	mouse	Forward: 5'-ACCTTTGCCGCTCATTTTCC-3'<br>Reverse: 5'-GCTGCACACACTCTTTACCG-3'
Tbx1	mouse	Forward: 5'-GGCAGGCAGACGAATGTTC-3'<br>Reverse: 5'-GCCGTGTTAAGGAATCTG CTG-3'
Prdm16	mouse	Forward: 5'-CAGCACGGTGAAGCCATTC-3'<br>Reverse: 5'-GCCGTGTTAAGGAATCTG CTG-3'
Pgc-1a	mouse	Forward: 5'-AGCCGTGACCACTGACAAC GAG-3'<br>Reverse: 5'-GCTGCATGGTTCTGAGTGCTAAG-3'
CKmt2	mouse	Forward: 5'-GCATGGTGGCTGGTGATGAG-3'<br>Reverse: 5'-AAACTGCCCGTGAGTAATCTT G-3'
Espt1	mouse	Forward: 5'-ACCCTGATAGCACCAAACGA-3'<br>Reverse: 5'-AGGTCTGCCAGTTCTTGCTC-3'
Tmem26	mouse	Forward: 5'-ACCCTGTCATCCCACAGAG-3'<br>Reverse: 5'-TGTTTGGTGGAGTCCTAAFFTC-3'
Cpt1	mouse	Forward: 5'- TTGCCCTACAGCTCTGGCATTTCC-3'<br>Reverse: 5'- GCACCCAGATGATTGGGATACTGT-3'
Cox4β	mouse	Forward: 5'- CTGCCCGGAGTCTGGTAATG-3'<br>Reverse: 5'- CAGTCAACGTAGGGGGTCATC-3'
CytC	mouse	Forward: 5'- AAATCTCCACGGTCTGTTCGG-3'<br>Reverse: 5'- GGGTATCCTCTCCCCAGGTG-3'
Mcad	mouse	Forward: 5'- ATGACGGAGCAGCCAATGAT-3'<br>Reverse: 5'- TCGTCACCCTTCTTCTCTGCTT-3'

Supplemental Table 2. RNA-seq analysis of the expression of G_s-linked GPCRs expressed in mouse inguinal white adipocytes (iWAT), epididymal white adipocytes (eWAT), and brown adipose tissue (BAT)

GPCR	Full name	G protein coupling profile	Reads Per Kilobase of transcript, per Million mapped reads (RPKM)
Adcyap1r1	adenylate cyclase activating polypeptide 1 receptor type 1	G_s, G_q/G_{11}	iWAT (1.083), eWAT (3.009), BAT (0.926)
Adora2a	adenosine receptor A2a	G_s, G_q/G_{11}	iWAT (2.602), eWAT (3.666), BAT (3.872)
Adora2b	adenosine receptor A2b	G_s, G_q/G_{11}	iWAT (0.230), eWAT (0.656), BAT (0.030)
Adrb1	adrenergic receptor, beta 1	G_s, G_i	iWAT (1.309), eWAT (0.701), BAT (8.282)
Adrb2	adrenergic receptor, beta 2	G_s, G_i	iWAT (2.200), eWAT (2.734), BAT (1.325)
Adrb3	adrenergic receptor, beta 3	G_s, G_i	iWAT (93.89), eWAT (147.456), BAT (71.26)
Avpr2	arginine vasopressin receptor 2	G_s	iWAT (0.105), eWAT (0.126), BAT (0.137)
Drd1a	dopamine receptor D1	G_s	iWAT (0.294), eWAT (1.083), BAT (0.017)
Fzd3	frizzled class receptor 3	G_s	iWAT (0.711), eWAT (0.696), BAT (0.129)
Gcgr	glucagon receptor	G_s	iWAT (0.081), eWAT (0.230), BAT (0.033)
Gipr	gastric inhibitory polypeptide receptor	G_s	iWAT (0.275), eWAT (0.566), BAT (0.426)
Gpr133	adhesion G protein-coupled receptor D1	G_s	iWAT (0.162), eWAT (0.379), BAT (0.177)
Gpr3	G protein-coupled receptor 3	G_s	iWAT (0.055), eWAT (0.050), BAT (0.011)
Gpr65	G protein-coupled receptor 65	G_s	iWAT (0.257), eWAT (1.313), BAT (0.603)
Htr7	5-hydroxytryptamine (serotonin) receptor 7	G_s	iWAT (0.015), eWAT (0.051), BAT (0.028)
Mc1r	melanocortin 1 receptor	G_s	iWAT (0.022), eWAT (0.014), BAT (0.006)
Mc2r	melanocortin 2 receptor	G_s	iWAT (8.250), eWAT (9.427), BAT (0.607)
Mc5r	melanocortin 5 receptor	G_s	iWAT (0.389), eWAT (0.173), BAT (0.416)
Ptger2	prostaglandin E receptor 2 (subtype EP2)	G_s	iWAT (0.111), eWAT (0.276), BAT (0.232)
Ptger4	prostaglandin E receptor 4 (subtype EP4)	G_s, G_i	iWAT (1.880), eWAT (2.831), BAT (0.839)
Pth1r	Parathyroid hormone 1 receptor	G_s, G_q/G_{11}	iWAT (2.479), eWAT (2.830), BAT (7.983)
Tshr	thyroid stimulating hormone receptor	G_s, G_q/G_{11}	iWAT (34.19), eWAT (36.89), BAT (2.395)
Vipr1	vasoactive intestinal peptide receptor 1	G_s	iWAT (0.010), eWAT (0.042), BAT (0.010)
Vipr2	vasoactive intestinal peptide receptor 2	G_s	iWAT (0.076), eWAT (0.182), BAT (0.124)

Adipocytes/BAT were isolated from 16-week-old male C57BL/6J mice consuming regular chow (n=6). RPKM data are given as mean values (n=6).
